# Recruiting South Asians to a lifestyle intervention trial: experiences and lessons from PODOSA (Prevention of Diabetes & Obesity in South Asians)

**DOI:** 10.1186/1745-6215-12-220

**Published:** 2011-10-06

**Authors:** Anne Douglas, Raj S Bhopal, Ruby Bhopal, John F Forbes, Jason MR Gill, Julia Lawton, John McKnight, Gordon Murray, Naveed Sattar, Anu Sharma, Jaakko Tuomilehto, Sunita Wallia, Sarah H Wild, Aziz Sheikh

**Affiliations:** 1 Centre for Population Health Sciences, The University of Edinburgh, Medical School, Teviot Place, Edinburgh EH8 9AG, UK; 2 Institute of Cardiovascular and Medical Sciences, University of Glasgow, Glasgow, G12 8QQ, UK; 3 Metabolic Unit, Anne Ferguson Building, Western General Hospital, Crewe Road, Edinburgh EH4 2XU, UK; 4 South Ostrobothnia Central Hospital, 60220 Seinäjoki, Finland; 5 Red RECAVA Grupo RD06/0014/0015, Hospital Universitario La Paz, 28046 Madrid, Spain; 6 Centre for Vascular Prevention, Danube-University Krems, 3500 Krems, Austria

## Abstract

**Background:**

Despite the growing emphasis on the inclusion of ethnic minority patients in research, there is little published on the recruitment of these populations especially to randomised, community based, lifestyle intervention trials in the UK.

**Methods:**

We share our experience of recruitment to screening in the PODOSA (Prevention of Diabetes and Obesity in South Asians) trial, which screened 1319 recruits (target 1800) for trial eligibility. A multi-pronged recruitment approach was used. Enrolment via the National Health Service included direct referrals from health care professionals and written invitations via general practices. Recruitment within the community was carried out by both the research team and through our partnerships with local South Asian groups and organisations. Participants were encouraged to refer friends and family throughout the recruitment period.

**Results:**

Health care professionals referred only 55 potential participants. The response to written invitations via general practitioners was 5.2%, lower than reported in other general populations. Community orientated, personal approaches for recruitment were comparatively effective yielding 1728 referrals (82%) to the screening stage.

**Conclusions:**

The PODOSA experience shows that a community orientated, personal approach for recruiting South Asian ethnic minority populations can be successful in a trial setting. We recommend that consideration is given to cover recruitment costs associated with community engagement and other personalised approaches. Researchers should consider prioritising approaches that minimise interference with professionals' work and, particularly in the current economic climate, keep costs to a minimum. The lessons learned in PODOSA should contribute to future community based trials in South Asians.

**Trial Registration:**

Current Controlled Trials ISRCTN25729565

## Background

There is insufficient participation of ethnic minority populations in complex research such as controlled trials and cohort studies particularly in the UK and other parts of Europe, and to a lesser extent in the USA [1-4]. This is unacceptable on ethical, scientific and policy grounds [5,6]. While theory and principles on how to recruit ethnic minority groups into trials are accumulating [7], reported experience outside of the USA is rare, especially in community based trials in Europe. The Scottish Ethnicity and Health Research Strategy [8] and a review by Netto *et al *[9] pinpointed the dearth of evidence for preventive trials in ethnic minorities in the UK with none in Scotland.

In this paper, we share our experiences of enrolling participants to the screening stage of the PODOSA trial and informally compare referral rates from the different approaches used. We have also attempted to judge the efficiency of the methods adopted. The lessons learned may help inform the development of future trials of interventions to ethnic minority populations in the UK, particularly in relation to recruitment approaches. We begin by providing an overview of published experience of recruiting South Asians into community based studies.

### Overview of recruitment strategies

There is some published experience in the UK, on recruitment of ethnic minority populations into descriptive and qualitative research, including studies on diabetes and its risk factors [10,11]. The main lessons from this growing body of research suggest that a multi-pronged approach should be adopted [12,13] and that face to face recruitment approaches tend to be most successful [10,14]. This has also recently been highlighted by Rooney et al who explored views about recruitment amongst South Asians with asthma. The authors suggest that successful recruitment necessitates using more resource intensive and personalised approaches than are commonly applied in the White European origin population [15]. There is also evidence that ethnically matched researchers have more understanding and empathy towards the participant's situation, culture and experience compared to non-matched researchers [16,17]. This is more likely to lead to establishment of trust which itself may contribute towards a willingness to take part in research.

However, there is little published evidence on recruiting ethnic minorities into health-related, community-based randomised trials, which require much larger participant numbers and arguably a much greater level of participant commitment than is required for qualitative research. Hussain-Gambles' review of the literature [18] revealed the paucity of ethnic recruitment into trials in the UK and explored attitudes to clinical trials [19]. Most community trials have been conducted in the USA [7,20]. Recruitment via existing participants, primary care providers, community clinics and providers have been highly recommended by Swanson and Ward based on largely USA experience [12]. A systematic review in 2006 of strategies to recruit under-represented populations into cancer clinical trials concluded that available evidence was limited and did not allow generalisability amongst different minority groups [21]. At the time of writing we are unaware of published methodological experience describing recruitment of UK South Asians into community based trials.

## Methods

### Trial setting and design

The age-standardised prevalence rate of type 2 diabetes in South Asians in the UK is about four times higher than in the population as a whole. South Asians tend to be diagnosed with diabetes at a younger age and are more likely to suffer complications than White Europeans [22,23]. Other trials have shown that maintaining healthy weight and practising sufficient physical activity can prevent or delay the onset of type 2 diabetes in people at high risk of developing the disease [24-26]. However, the effect of lifestyle interventions has not been tested amongst South Asians living in the UK.

PODOSA is a family orientated, home-based, cluster randomised, lifestyle-intervention trial for South Asians (of Indian or Pakistani origin) who are 35 years or over living in NHS Lothian and Greater Glasgow & Clyde Health Board areas [27]. Eligible participants are those with impaired glucose regulation, either impaired fasting glucose (IFG) and/or impaired glucose tolerance (IGT), both conditions being linked to a high risk of developing type 2 diabetes and cardiovascular disease [28,29]. The primary aim of the trial is to test whether consulting with a trained dietitian over three years will achieve weight loss, an increase in physical activity and, in the longer term, prevention of type 2 diabetes. The main features of the trial are in Table 1. PODOSA's key innovations are to recruit Indian and Pakistani populations, to change the prevention intervention from the clinic to a home based setting, and to focus on the family and not just the individual. The intervention will finish in October 2012.

**Table 1:** Eligibility and outline of methods for PODOSA

Eligibility	Exclusion criteria	Methods
Pakistani/Indian origin	Current steroid medication	Participants are screened by an oral glucose tolerance test to identify those with impaired glucose tolerance (IGT) and/or impaired fasting glycaemia (IFG)

Living in Edinburgh or Glasgow areas	Pregnancy	Recruits with IGT/IFG plus any family volunteers are randomised into two groups, one group having 15 dietitian contacts, the other 4 contacts, over 3 years

Age ≥ 35 years	Expectation of emigration, or a medical condition indicating adherence to the study intervention would be unlikely	Dietitians visit the participating families in their homes, to provide advice and motivational support in relation to losing weight and increasing physical activity

Waist ≥ 90 cm (35in) for men and ≥ 80 cm (31in) for women		

### Initial recruitment expectations and strategies

The initial strategy for PODOSA recruitment involved, as recommended by the published literature, a range of approaches; these are detailed below.

1. Secondary/primary care referral from the National Health Service

2. Responses to written invitations to potential participants sent via General Practitioners (GPs) & Lothian and Glasgow Diabetes registers

3. Personal contacts of the study team and snowball effect i.e. recruited participants enlisted others

4. Local South Asian organisations' and their leaders' referrals

5. Media promotion, posters/leaflets, website, radio etc leading to self-referral

Based on 2001 Scottish census data and findings from Newcastle upon Tyne, we estimated that our target population would comprise at least 17,000 South Asians over 35 years of age, of who around 4000 might have IGT [11]. As recommended by Hussain-Gambles [30], during the design stage, we informally discussed the proposed trial with health care professionals and some South Asian community members, as well as involving collaborators who were closely engaged with the South Asian community. A pilot study was not undertaken, partly because this would have required substantial resources and time and partly because of the existing international experience from the major diabetes prevention studies [24-26]. Furthermore, members of the research team were experienced in recruiting South Asians to cross-sectional studies with relatively high response rates [11]. We employed three South Asian bilingual dietitians, two with extensive work experience in Glasgow. Professional and clinical experience had taught us that this was important, as a significant proportion of the South Asian population in the two cities, especially in the older age groups, speak and read little English. Ethical approval was obtained from Scotland Multi-centre Research Ethics Committee who directed us to concentrate on recruitment via the NHS and only if necessary to recruit directly from community-based sources. On the basis of the preparatory work and due to type 2 diabetes being a major health issue within the South Asian population in Scotland, we anticipated the public and professional enthusiasm for the trial would be translated into demand for participation. We initially aimed to recruit around 50% of participants for screening via the health service (NHS) (1-2 above) and the remainder via community approaches (3-5 above). However, our target of screening 1800 South Asians with an oral glucose tolerance test in 10 months was unachievable because recruitment proved difficult and because this ambitious target had been based on clinic centred experience from the Newcastle Heart Project [11].

### Summary of our actual experience of recruitment

Recruitment to PODOSA commenced in July 2007 and closed in October 2009. Although we introduced all strategies from the outset of recruitment, we initially focussed on the health service approach as directed by the ethics committee. As detailed below, we promoted the trial widely. As in the preparatory phase, we found there was widespread approval of, and support for, the aims of the trial amongst both health care professionals and community leaders. However it quickly became apparent that the level of recruitment via NHS channels, was going to be much lower than the rate originally hoped for. In response, we increased direct promotional and recruitment efforts within the South Asian communities. After the initial nine months of recruitment we had screened around 400 people compared to the target of 1600. Experience from the research team's promotional talks and visits within the community showed that face-to-face recruitment either individually or with small groups was relatively successful. However we found that the goodwill of local community groups and organisations to help with recruitment was insufficient for a project of this scale. Several organisations were keen to assist, but had limited resources to allow staff to spend time actively recruiting for PODOSA.

Therefore in March 2008, we sought and acquired new funding and subsequently partnered with five local organisations and 10 individuals to recruit for us. Our agreement with these groups and individuals was to pay £15 per person referred who was subsequently screened. At this time we also initiated a similar payment scheme for general practitioners who referred patients to the study. Towards the end of 2008 we contracted with a marketing and consultancy company, specialised in working with the South Asian community, to adapt our materials and to market PODOSA.

Table 2 summarises the source and numbers of referrals achieved in relation to initial targets. The main criteria for referrals were South Asians of Indian- or Pakistani-origin, aged 35 years or over and without diabetes. The dietitans then had to assess potential participants for eligibility to be screened, including waist size, availability for the three year intervention period, and clinical exclusion criteria. The approaches are set out below, with numbering corresponding to that in Table 2. (We did not record the total number of contacts made informally with potential recruits by our research dietitians or by the community recruiters.)

**Table 2:** Recruitment strategies to identify participants for screening stage of PODOSA

Source	*No. of referrals/responses (% of total)	Initial target (%) for screening participants by source	^+^% of total actually screened (estimated)	Judgement on success of strategy
**1. NHS**				

1 (a) Direct referrals from health care professionals	55 (3)	25	1	Largely unsuccessful

1 (b) Written invitations via GPs to potential participants	265 (13)	25	11	Low (5.2%) response rate to letters was resource intensive

1 (c) Written invitation via diabetes register to diabetes patients (to target their relatives)	16		0	Unsuccessful

1 (d) Search of practice lists for IGT/IFG	4		0	Unsuccessful

**Subtotal**	**336 (16)**	**50**	**12**	**Limited success**

**2. Community**				

2 (a) Via research team contacts, self referrals and 'snowball' effect	> 630 (30)		47	Successful particularly in Glasgow, at minimal cost

2 (b) Community organisations and recruiters, assisting with recruitment for small payment	618 (29)	(a), (b) and (c) 50	26	Initially unsuccessful when relying on goodwill, moderately successful when payment offered

2 (c) Research team recruitment via visits/talks	480 (23)		14	Moderately successful but labour intensive

**Subtotal**	**1728 (82)**	**50**	**87**	**Successful**

**3. Media techniques**				

3 (a) Written articles in the press, radio interviews, leaflet and poster distribution, website and e-mail distribution lists	Exact number not known, but few	Mainly to raise awareness with the expectation of some self-referrals	0	Not successful in directly enrolling participants

3 (b) Ethnic marketing and consultancy company	25 (1)		1	Limited success achieved by fieldwork, not mass marketing

**Subtotal**	**> 25 (2)**	**-**	**1**	**Unsuccessful**

**Totals**	**> 2089 (100)**	**100%**	**100% (1319)**	

* total number of potentially suitable participants referred to research team or responded to invitation letters for the screening stage

^+ ^1319 of 2089 referrals were eligible, available and willing to attend a screening visit to have blood glucose measured, percent of total screened is given

1 (a) *Direct NHS referrals*

Before and during recruitment, we promoted PODOSA to professionals in both primary and secondary care via presentations and face-to-face discussions. Study information leaflets in English, Punjabi and Urdu, posters and referral forms were distributed to general practices and secondary care diabetes clinics in each city. The aim was to raise awareness of the study with the expectation that health care professionals would refer potentially eligible patients to the research team.

1 (b) *NHS: written invitations via General Practices*

Fifteen practices in Glasgow and 18 in Edinburgh were identified as having the largest number of South Asian patients. Practice lists were searched for patients aged 35 years or older without a diagnosis of diabetes and then scanned for common South Asian surnames. The ensuing lists were checked by practice staff prior to these patients being invited. Personally addressed invitation letters with study information leaflets in three languages and reply forms were sent from the practice. Interested participants were given the option to respond by pre-paid mail, phone or email.

1 (c) *Written invitations via the diabetes register*

NHS Lothian and Greater Glasgow & Clyde have diabetes registers with levels of ethnicity coding exceeding 60%. In Lothian, we approached South Asians already diagnosed with diabetes to seek participation of their relatives, who might be especially receptive to the idea of a prevention programme. General practices, as the owners of the diabetes register data, agreed to their patients being sent a recruitment pack to pass onto family members and friends.

1 (d) *Search of practice lists for IGT/IFG*

We piloted an electronic search of the practice lists of six general practices using READ codes (the coding system used in UK general practice) to identify potentially eligible patients who already had a recorded diagnosis of IGT or IFG. The aim was to target our specific study population as outlined in Table 1.

All patient searches described above were carried out by either primary care or diabetes network staff who had the authority and relevant approvals to access patient records.

2 (a) *Community recruitment: research team contacts and the snowball effect*

Personal contacts provided us with links to numerous local community leaders and groups. During the initial recruitment period one dietitian in Glasgow enrolled three individuals into the screening stage, one from each of the Sikh, Hindu and Muslim faiths. During the first nine months, 140 further participants were screened for the study (34% of the total screened at that point) as a result of snowballing via these three initial contacts. The dietitians asked all those attending the screening visit if they had family or friends who might be interested in participating and if appropriate gave them a supply of study information leaflets to pass on.

2 (b) *Community recruitment: using community and faith organisations*

We had support from community leaders, including many of the faith organisations in both cities, and other influential people, e.g. the Indian and Pakistani Consuls, a Member of Parliament, and other leaders. Many community and religious organisations were approached by the research team, the intention being to carry out initial promotional talks ourselves, then to ask these groups to pass on information to their clients and members.

After securing additional funding, we set up formal partnerships with five local organisations, including: NHS or community health initiatives, a women and children's Islamic teaching organisation and, the Muslim Council for Scotland, a national body to promote Muslim affairs in Scotland. We also identified 10 individual recruiters who were well known within their local communities. Contracts were agreed with the groups and individuals, based on a payment of £15 per referral actually screened. All the paid recruiters were given materials and training about diabetes, the risk for South Asians, the trial eligibility criteria and the importance of confidentiality.

2 (c) *Research team's visits/talks*

During the 27 month recruitment period, the research team gave over 60 talks in a range of community organisations including many temples and mosques and at local South Asian events. The talks focussed on South Asians' risk of developing type 2 diabetes, how it can be prevented and what trial participation would involve. We also attended melas (South Asian fairs) and other such gatherings.

3 *Media promotion*

3(a) PODOSA published in NHS, local and South Asian specific newspapers. The trial was promoted in the Indian and Pakistani communities through poster and leaflet distribution. Our information leaflet used simple language to describe the study, explain that South Asians are at high risk of developing diabetes, and provide the research team's contact details. It was translated into Urdu and Punjabi and all language versions were tested for understanding within the community, using local contacts conversant in these languages. The study website contained information about the trial, prevention of diabetes, and had a simple self-referral registration facility. Other methods utilised were e-mails to distribution lists of various South Asian organisations and well connected individuals, and publicity via interviews on the Glasgow South Asian Radio station (Radio Awaz). The main aim was to raise awareness but with an expectation that this would lead to some self-referrals.

3(b) *Marketing Agency*

A marketing and promotions company designed an e-flyer and new promotional poster to a high professional standard, and initiated a marketing campaign. This involved regular email-shots to their large database of South Asian contacts, widespread poster distribution and access to local media, for example arranging radio interviews with the Principal Investigator.

## Results

### Recruitment from the Health Service

1 (a) Despite targeting practices with large South Asian populations and actively seeking involvement of their general practitioners, as well as introducing a small financial incentive from June 2008, we received only 55 direct referrals from health care professionals during the 27 month recruitment period. There was no increase in referral rate following the introduction of the small financial reimbursement. Only 30% of these referrals were eligible and available to attend for study screening. Table 2 shows that this method led to 1% of the total actually screened whereas 25% had originally been aimed for.

1 (b) The response rate from the written invitations via general practices was 5.2% (265 responses from 5071 invitations). This approach was carried out over a period of ten months and contributed 11% of the 1319 participants who were screened. Reminder letters to non-responders were piloted in two practices with a 3% response rate. We judged that it was not cost-effective to continue this process.

1 (c) The indirect approach via South Asian patients on the Lothian diabetes register resulted in a response rate of 4.2% (16 responses from 378 letters). In light of our experience in Lothian, the Glasgow register was not used.

1 (d) Only eight individuals were identified from the search of six general practice lists for IGT/IFG of whom four were South Asian and over 35 years of age.

Overall, participants identified via the health service contributed 12% of study recruits actually screened as shown in Table 2.

### Community recruitment

2 (a) Overall, direct recruitment via research team contacts and referrals from participants themselves (snowballing) provided a significant proportion (at least 30%) of referrals to PODOSA.

2 (b) The paid community recruiters found the recruitment process harder than anticipated. Five of the 10 individuals did not refer any participants. The recruiters' main reasons for this were a lack of time and limited access to the relevant population of South Asians living permanently in Glasgow or Edinburgh. As Table 2 shows, in total the community recruiters contributed approximately 29% of the referrals to PODOSA. Of the 618 names passed to the research team, collected over a period of about 18 months, around 55% were eligible, available and willing to be screened.

2 (c) The research team's efforts resulted in the collection of 480 names of potential participants (23% of total). It was a resource intensive strategy with variable success. Fifty names were collected within an hour at a visit to one mosque but other talks (including at other mosques) resulted in only a handful of people coming forward and took up many hours of the research team's time.

### Media promotion

3 (a) and 3 (b) Table 2 shows that there was minimal direct response to these approaches. The marketing campaign resulted in only 25 additional known referrals, mostly from face-to-face recruitment carried out by the agency.

### Recruitment into the trial

**Figure 1:**
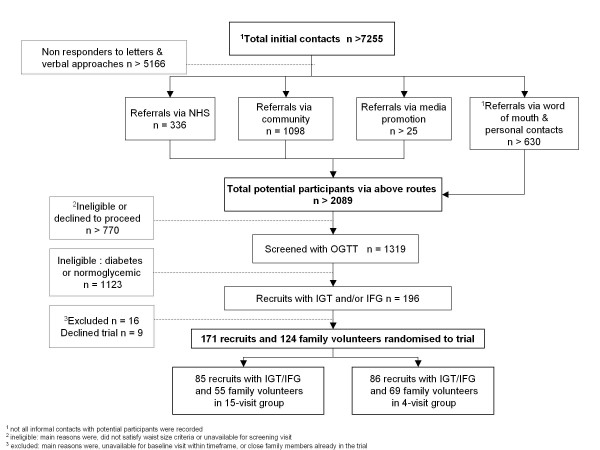
**Recruitment flow chart**.

Overall, we screened 1319 recruits in 27 months as shown in Figure 1 and randomised 171 recruits with IGT/IFG into the trial. In contrast with recruitment to screening, once participants had been identified with dysglycaemia, 95% (171 of 180) agreed to take part in the three year trial.

## Discussion

The most successful recruitment strategies into PODOSA were the partnerships with the local South Asian organisations and individuals, and referrals by word of mouth from existing participants, contributing 59% of the total referrals and providing a low cost source of recruitment. In turn this led to 73% of the total number screened. Of the 2089 people referred for screening 1319 were eligible, available and agreed to be screened. Around 30% of the GP referrals and 55% of those referred by the community recruiters were screened. This difference is probably due to the more direct training and closer communication about eligibility given to the community recruiters. The research dietitians estimated that a much higher percentage of self-referrals via snowballing were eligible to proceed to the screening stage, which further adds to the success of this particular method.

Our response rate of 5% to written invitations via general practices is lower than the 22% rate reported in studies involving general populations in central Scotland using a similar written approach [31,32]. These two studies involved an older population (aged over 50 years) than ours which may account towards some of the difference. A lifestyle intervention study for individuals with increased risk of coronary heart disease living in Paisley, Scotland, invited people aged 45-60 for eligibility screening. The researchers reported a 16% response to mass mailings alongside media promotion and awareness raising events [33]. The ProActive trial, aimed at people aged 30 - 50 years in South-east England [34], achieved 67% and 77% overall response rates via primary care diabetes and family history registers respectively, considerably higher than our rate of 5%. Invitation letters were targeted at the offspring of diabetes patients, which is a similar approach to ours using the diabetes registers, but, in our case, such an approach only yielded a 4.2% response. The inclusion of a reminder letter and asking the diabetes patients to respond with the contact details for their family, may have partly contributed to the higher response rates in the ProActive trial.

In addition, in PODOSA, it was difficult to identify South Asian participants in the absence of ethnic coding in the record systems, which may partly explain why recruitment via the health service (NHS) proved to be an inefficient process, particularly when it is compared to other trials and studies which are not restricted to South Asian populations.

However our response rate was similar to that achieved in the Bangladip study in Tower Hamlets, London which also involved a South Asian (Bangladeshi) population (G Hitman, 2010, personal communication). This would suggest that South Asians are less inclined than the general population to respond to a written approach, even when high quality translated materials are used as happened in the PODOSA trial. Further research to understand why this is so would be valuable.

Recruitment via patients on the diabetes registers was time consuming, requiring collaboration between the research team and health service staff with access to register data and the general practices, and gaining permissions.

It proved difficult for health care professionals to find time in their day-to-day practice to identify potentially eligible patients and explain the study and the referral process to them. The introduction of a small reimbursement, part way through the recruitment period, had no effect on referral rate. Future studies may wish to consider larger payments as an incentive to encourage referrals from primary care. A questionnaire survey [35] examining recruiters' experiences when enrolling participants for PODOSA reported that the main reasons for unsuccessful referrals from health professionals were: lack of time on their part, and lack of interest in and/or understanding of the implication of diabetes and research on the part of their patients. Similar experiences have been reported elsewhere [36-38].

Our electronic search of practice lists for South Asians with IGT and/or IFG identified so few (four) potentially eligible patients that this was not considered a worthwhile approach to use while levels of recording of pre-diabetes, and of ethnicity, in primary care remain so low. Overall, recruitment via the health service had limited success and was time and resource intensive. Ethics Committees should note this for future trials, and may wish to encourage early direct contact for community recruitment [39] as part of a multi pronged recruitment program tailored to the ethnic minority group under study.

Media promotion probably raised awareness of the study but, as shown in Table 2, there was minimal direct response to these approaches. We judge, however, that there is value in working with a media company prior to recruitment commencing, as they can provide specific expertise in a targeted promotion campaign using their wide network of contacts.

### Limitations

PODOSA was not set up to collect detailed data on resources, time and costs for the recruitment process and therefore to formally compare each strategy. We acknowledge this as a limitation to the conclusions that can be drawn from our findings. However we have formed a judgement on the success of the different approaches based on numbers referred, and informal estimates of time and cost incurred by the research team, staff from other research networks and community recruiters.

## Conclusions

We faced many challenges during the initial recruitment stage to PODOSA of the kind that have delayed or stopped other trials in general populations [40]. As reports of working with South Asian populations in the community in the UK are limited, we hope our experience will help others and provide encouragement for further studies to be undertaken in this and other UK ethnic minority populations. As others have suggested [21,41], making generalisations for recruitment to other ethnic minority groups may not be valid, however improving understanding in one population may help raise awareness of possible recruitment issues for community trials in other minority groups.

Within PODOSA, recruitment via the health service was not efficient or sufficiently effective, and other methods needed to be used. Community involvement worked well. PODOSA demonstrates that these methods, which have been used in qualitative studies [10,19], also succeed in the context of a randomised trial. Our greatest lesson is that the costs of direct community recruitment in partnership with local organisations need to be included as a grant cost. This agrees with Stirland et al's findings for recruitment of South Asians into asthma research [42]. It was naive of us to assume that public and professional enthusiasm for the trial, and the widely accepted need to tackle diabetes in South Asians living in Scotland, would, in themselves, rapidly lead to referral or self-referral. Piloting recruitment methods (rather than the whole trial) should be considered by future research teams but is not easy in the context of a multi-pronged enrolment strategy. Also, a pilot study might not yield the results of the actual trial.

In the current economic climate, where value for money is a key criteria for funders, researchers will need to adopt approaches that are cost-efficient and do not interfere with professionals' work. We propose that our experiences will be of value, both for informing further research on this issue, and for making pragmatic decisions about recruitment of South Asians to clinical trials pending availability of further evidence.

## Competing interests

The authors declare that they have no competing interests.

## Authors' contributions

D is the lead writer and trial manager, B is PI and co-author, W, B (Ruby), and S are research dietitians carrying out the study recruitment, screening and interventions. B, W, F, G, McK, M, S, T, W, L, S and D helped plan the trial. All authors researched data and contributed to critically revise successive drafts of the manuscript. All authors read and approved the final manuscript.
